# Regulating autophagy facilitated therapeutic efficacy of the sonic Hedgehog pathway inhibition on lung adenocarcinoma through GLI2 suppression and ROS production

**DOI:** 10.1038/s41419-019-1840-6

**Published:** 2019-08-19

**Authors:** Jiajun Fan, Xuyao Zhang, Shaofei Wang, Wei Chen, Yubin Li, Xian Zeng, Yichen Wang, Jingyun Luan, Li Li, Ziyu Wang, Xilin Sun, Baozhong Shen, Dianwen Ju

**Affiliations:** 1 Minhang Hospital, Fudan University, 170 Xinsong Road, Shanghai, 201199 China; 2 Department of Microbiological and Biochemical Pharmacy and The Key Laboratory of Smart Drug Delivery, Ministry of Education, School of Pharmacy, Fudan University, Shanghai, 201203 China; 3 The Fourth Hospital of Harbin Medical University, Harbin, 150028 P. R. China

**Keywords:** Cancer therapy, Medical research

## Abstract

Lung adenocarcinoma (LUAD), which comprises over 50% of all cases of non-small-cell lung cancer, has a poor prognosis and requires novel therapeutic approaches. The sonic Hedgehog (Shh) pathway, which plays a crucial role in differentiation, proliferation, and survival of cancer cells, is likely to be activated in LUADs, suggesting the Shh pathway as a potential therapeutic target for LUAD treatment. In this study, we reported that vismodegib, an inhibitor of the Shh pathway, only elicited minor antitumor efficacy in A549 and NCI-H1975 LUAD cells as well as in the xenograft tumors, with overexpressed GLI2 and increased autophagic activity. The aberrant autophagy in LUAD cells was further confirmed by the three main stages of autophagic flux, including the formation of autophagosomes, the fusion of autophagosomes with lysosomes, and degradation of autophagosomes in lysosomes. Furthermore, inhibition of autophagy by siRNA against ATG5 or ATG7 rescued the sensitivity of A549 and NCI-H1975 LUAD cells to vismodegib in vitro. Meanwhile, administration of the pharmaceutical inhibitor of autophagy, chloroquine, contributed to the enhanced anti-LUAD efficacy of vismodegib in vivo, probably through overproduction of ROS, acceleration of apoptosis, and suppression of GLI2 in LUAD tissues. In summary, our research revealed that downregulating autophagy facilitated the anti-LUAD efficacy of the Shh pathway suppression, thus highlighting a potential approach for LUAD therapy via simultaneously targeting the Shh signaling and autophagy pathway.

## Introduction

Lung adenocarcinoma (LUAD), which accounts for ~50% of non-small-cell lung cancer (NSCLC), has become one of the leading causes of cancer-related mortality worldwide^1^. Although the developed therapies such as platinum and radiotherapy have greatly benefited the management of lung cancer patients, the prognosis of LUAD patients still remains poor, with the 5-year survival rate at ~15%^2^. Therefore, novel approaches are urgently required for LUAD therapy.

Sonic Hedgehog (Shh) is an evolutionarily conserved signaling pathway essential for the development of early embryos^3^. Due to its crucial role in differentiation, proliferation, and survival of cancer cells, the Shh signaling pathway has been becoming one of the most promising targets for cancer therapy. Vismodegib (GDC-0449) is the first Shh inhibitor approved for the clinical treatment of basal cell carcinomas (BCC)^4,5^. It could selectively deprive the activity of the Smoothened (SMO) and finally result in the apoptosis induction and growth inhibition in BCC cells^6,7^. Indeed, vismodegib seemed to have a broad-spectrum growth inhibitory effect on tumor cells with constitutively active shh signaling. It was documented that inhibition of the Shh pathway could silence the activity of GLI1/2/3^8,9^ and thereby induced caspase-dependent apoptosis and growth inhibition in non-Hodgkin lymphoma cells and chronic myeloid leukemia cells^10,11^. Similarly, highly activated GLI1 and GLI2 were also observed in LUADs^12^, indicating that the signaling transduction of the Shh pathway was abnormally activated in LUADs, and the Shh inhibitors might be a potential therapeutics for LUAD therapy. However, our preliminary results showed that vismodegib only had minor effect on the LUAD cells with unknown mechanisms.

Autophagy is a basic catabolic process that involves degradation of unnecessary or dysfunctional cellular components through their recognition and uptake by autophagosomes and lysosomes^13,14^. When cells were under severe conditions, such as hypoxia, intracellular stress or nutrient deficiency, autophagy was usually promoted to maintain the microenvironment for cell survival^15–17^. Although the function of autophagy in tumor therapy still remained controversial, evidence showed that autophagy was a key regulator of the drug resistance during cancer therapy. It was reported that autophagy blockage by 3-methyladenine (3-MA) and bafilomycin A1 remarkably enhanced the gefitinib sensitivity in triple-negative breast cancer cells^18^, while AMPK-ULK1-mediated autophagy was demonstrated to confer the resistance to BET inhibitor JQ1 in acute myeloid leukemia stem cells^19,20^. Actually, regulation of the Shh signaling pathway is always related to the modulation of autophagy. Mounting evidence revealed that inhibition of the Shh signaling pathway by GANT61 and vismodegib could trigger cyto-protective autophagic flux in HepG2, Hep3B, K562, and Baf3-WT cells^11,21^, indicating relationship between induction of autophagy and acquirement of resistance against the Shh inhibitors. Therefore, we supposed that autophagy might act as a major player in vismodegib resistance during the treatment of LUAD.

In this work, we revealed that Shh inhibitor vismodegib exhibited only minor effect on A549 and NCI-H1975 LUAD cells in vitro and transplanted tumors in vivo. During this process, elevated autophagic flux was observed, which was likely to be a cyto-protective mechanism in response to vismodegib treatment. Importantly, we investigated whether and how autophagy participated in the resistance against vismodegib therapy in vitro and in vivo. Our results showed that inhibition of autophagy could promote the efficacy of vismodegib against LUAD, and thus highlighted a potential approach for LUAD therapy via simultaneous targeting Shh signaling and autophagy pathway.

## Results

### Unexpected anti-LUAD effect in vitro and in vivo after Shh signaling suppression by vismodegib

**Fig. 1:**
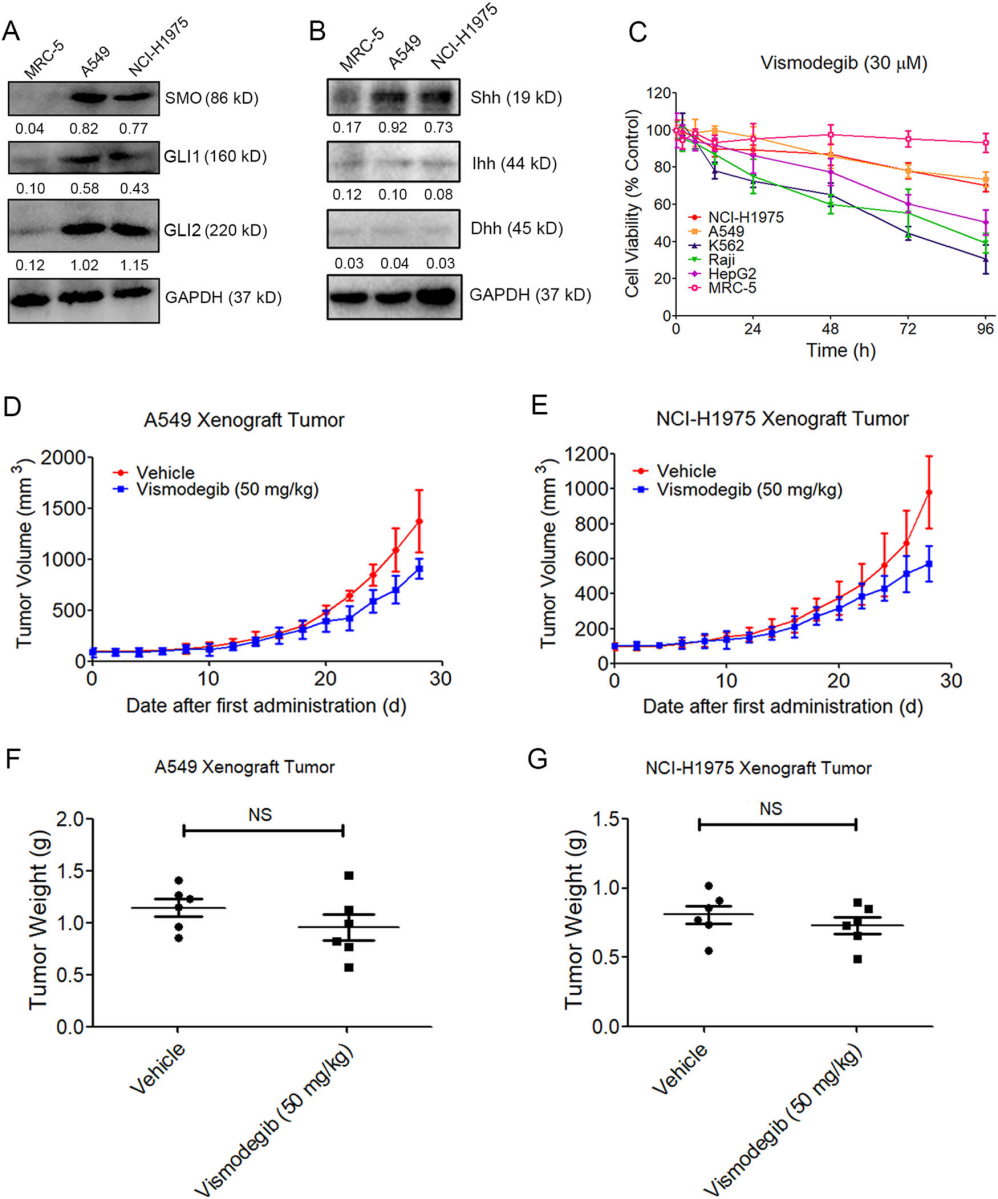
Limited anti-LUAD effect of vismodegib in vitro and in vivo. **a**, **b** Increased activity of the sonic Hedgehog signaling pathway in A549 and NCI-H1975 LUAD cells. Cell was lysed by RIPA, and western blot was applied to detect the expression of SMO, GLi1, GLi2, Shh, Ihh, and Dhh. The statistical analysis of each western blot is measured by being normalized to the level of GAPDH. **c** Cytotoxic effect of vismodeigb in Shh-overreacted cancer cells. **d**, **e** Tumor growth curve of A549 and NCI-H1975 xenograft tumor during 28 days after the vismodegib treatment. **f**, **g** No significant changes of tumor weight after mice treated with vismodegib. The Pharmacodynamic experiment to determine the anti-LUAD efficacy of vismodegib was repeated once (*n* = 6 for each group in every experiments), and the western blots as well as the MTT assays were repeated for three times

First, we assessed if the Shh signaling pathway was overactivated in LUAD. Western blot analysis showed that A549 and NCI-H1975 cells exhibited higher levels of SMO, GLI1, and GLI2 expression than the MRC-5 normal lung cells did (Fig. 1a, b). Besides, the SMO agonist SAG significantly enhanced the transcription of GLI1 (Fig. S1), indicating that the Shh singling pathway was likely to be activated in LUAD cells. Next, the cytotoxic effect of vismodegib on A549 and NCI-H1975 cells were determined in vitro. Unexpectedly, the vismodegib exhibited only a minor cytotoxic effect on A549 and NCI-H1975 cells, whereas it could efficiently kill the HepG2, Raji, and K562 cells (Fig. 1c), whose Shh signaling were also reported to be overactivated. Furthermore, we observed the limited inhibitory effect of vismodegib against the growth of the A549 and NCI-H1975 transplanted tumors in vivo (Fig. 1d–g). Thus, our data indicated the minor antitumor effects of vismodegib on LUADs.

**Fig. 2:**
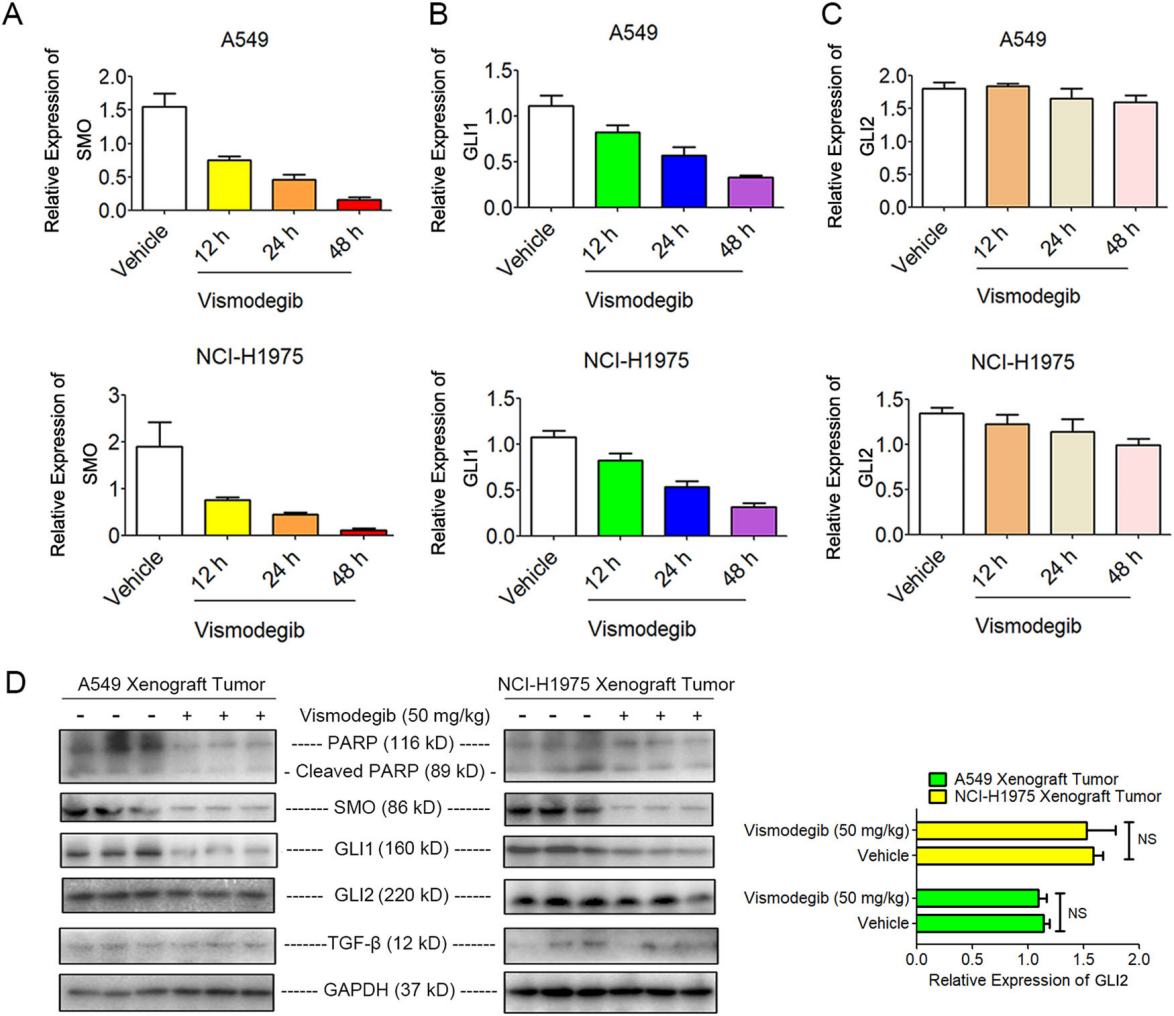
Reduced Level of GLI1 and SMO but no significant change of GLI2 in vitro and in vivo after vismodegib treatment. **a**, **b** Decreased mRNA levels of SMO and GLI1 in LUAD cells after vismodegib (30 μM) treatment. **c** No significant changes of GLI2 transcription in LUAD cells after vismodegib treatment. **d** Notable inhibitory effect on GLI1 and SMO but limited changes on cleavage PARP and GLI2 induced by vismodegib treatment in A549 and NCI-H1975 xenograft tumors. Both the RT-PCR and western blot analysis have been repeated for three times

To elucidate the underlying mechanism of minor antitumor effects of vismodegib therapy on LUADs, we tested the expression of Shh-related genes and proteins in LUAD cells or transplanted tumors after vismodegib treatment. Our data revealed that the mRNA levels of SMO and GLI1 were significantly reduced after vismodegib exposure, while the mRNA level of GLI2 only showed a minor change (Fig. S1A–C). Moreover, the expression levels of the Shh signaling pathway-related proteins were evaluated after the tumor-bearing mice were exposed to vismodegib for 28 days. Our results showed that vismodegib downregulated the expression of the SMO and GLI1 in A549 and NCI-H1975 transplanted tumors, but had no significant influence on that of GLI2 (Figs. 2d; S2), indicating that the inhibition of the SMO-related pathway individually could not potently kill LUAD cells. Furthermore, we also challenged if any factor such as TGF-β in the tumor microenvironment affected the transcription of GLIs. Our results showed that there was no significant change of TGF-β in LUAD tumors after mice were treated with vismodegib, indicating that the aberrant GLI2 expression was not due to the upregulation of TGF-β (Fig. 2d).

### Autophagy initiation and autophagic flux in A549 cells and NCI-H1975 cells after Shh signaling blockage

Since the activity of the Shh pathway is notably related to autophagy activity^22–25^, we detected whether autophagy was upregulated after vismodegib treatment. First, we determined the transcription of autophagy-related genes in vismodegib-treated A549 and NCI-H1975 cells. The mRNA levels of ATG5 and ATG7 have a 2.9-fold and 4.1-fold increase in A549 cells, as well as a 3.2-fold and 3.8-fold increase in NCI-H1975 cells after vismodegib treatment (Fig. S3A, B). Next, western blot analysis was employed to assess the expression of autophagic marker protein, type II microtube-associated protein light chain 3 (LC3-II). We found that LC3-II expression increased both in LUAD cells and in LUAD xenograft tumors after vismodegib treatment, (Fig. S4A–C), while 30 μM of vismodegib induced neither growth inhibition nor the remarkable upregulation of LC3-II level in HEK293 cells which lacked the functional Shh pathway (Fig. S5). These results indicated that formation of autophagic membrane in LUAD cells was probably due to the suppression of the Shh pathway. Furthermore, the results of fluorescent confocal microscopy also confirmed that cells treated with vismodegib showed more punctate fluorescence in plasma than the negative controls did after stained by Cyto-ID, an autophagosomes-specific green dye (Fig. S4D). Thus, our data demonstrated the formation of double-membraned vesicles when A549 and NCI-H1975 cells were exposed to vismodegib, indicating autophagy initiation in LUAD cells and xenograft tumors after vismodegib treatment.

**Fig. 3:**
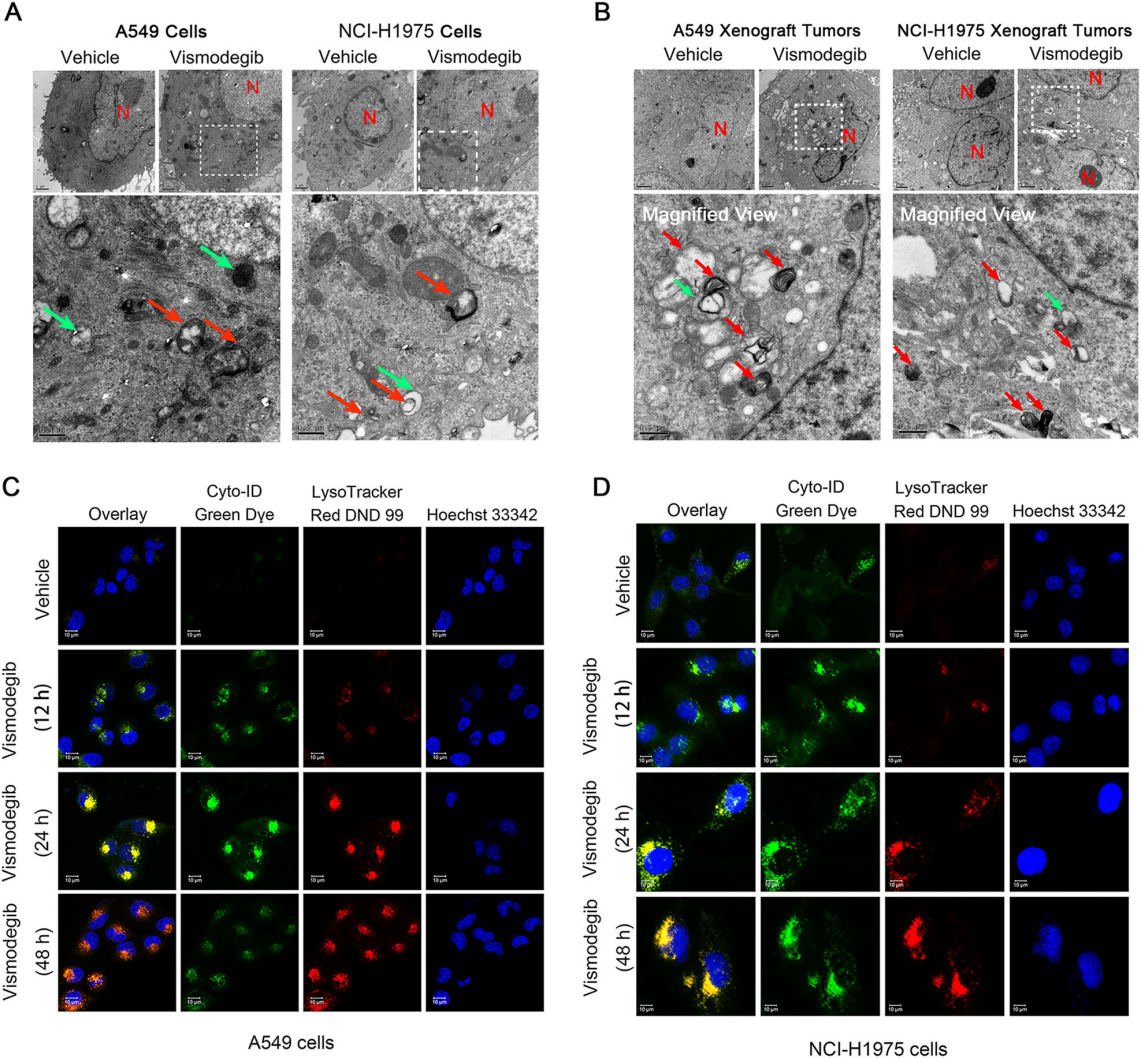
Autophagic flux in A549 and NCI-H1975 cells as well as in their transplanted tumors after vismodegib exposure. **a** Autophagosomes and autolysosomes accumulation in A549 and NCI-H1975 cells after vismodegib treatment. Cell were treated with vismodegib for 48 h and prepared for TEM analysis. The specific autophagosomes vesicles were marked as a red arrow, while the autolysosomes were marked as a green arrow (*N* = nuclear). **b** Autophagosomes and autolysosomes accumulation in A549 and NCI-H1975 transplanted tumors. Mice were killed after a 28-day treatment of vismodegib, and the tumor tissues were resected for TEM sample preparation immediately. The specific autophagosomes vesicles were marked as a red arrow, while the autolysosomes were marked as a green arrow (*N* = nuclear). **c**, **d** Autophagic flux in A549 and NCI-H1975 cells after vismodegib treatment. The autophagosomes were marked by cyto-ID dye, while the lysosomes were stained by lysotracker DND99. All the experiments have been repeated for three times

Importantly, autophagic flux was also observed after blocking the Shh pathway. The ultrastructure of autophagosomes (red arrow) and autolysosomes (green arrow) in A549 and NCI-H1975 cells were monitored by transmission electron microscope (TEM) after vismodegib exposure. As shown in Fig. 3a, vesicles with double membrane could be observed after cells treated with 30 μM of vismodegib, while the negative vehicle controls exhibited little double-membraned vesicles, indicating the accumulation of autophagosomes in vismodegib-treated A549 and NCI-H1975 cells. Interestingly, some particular single-membraned vesicles carrying the autophagosomes appeared in LUAD cells after vismodegib treatment, suggesting the autophagosomes fusing into lysosomes in vismodegib-treated LUAD cells. Similarly, these autophagic-specific ultrastructure could also be found in LUAD xenograft tumors from vismodegib-treated mice (Fig. 3b). Moreover, cyto-ID, the dye for autophagosomes, together with the lysotracker, was employed to evaluate autophagosomes formation and degradation in LUAD cells after vismodegib exposure. Importantly, three main stages of autophagic flux in vismodegib-treated cells could be observed under the confocal microscopy: autophagosomes accumulation at 12 h (green fluorescence), autophagosomes internalization into lysosomes at 24 h (green fluorescence co-localized with red fluorescence) and degradation of autophagosomes by lysosomes at 48 h (limited green fluorescence in red fluorescence) (Fig. 3c, d).

Taken together, our data suggested that complete autophagy was abnormally activated after shh signaling blockage in LUAD cells.

### Rescued sensitivity of A549 and NCI-H1975 cells to Shh signaling suppression after autophagy inhibition

**Fig. 4:**
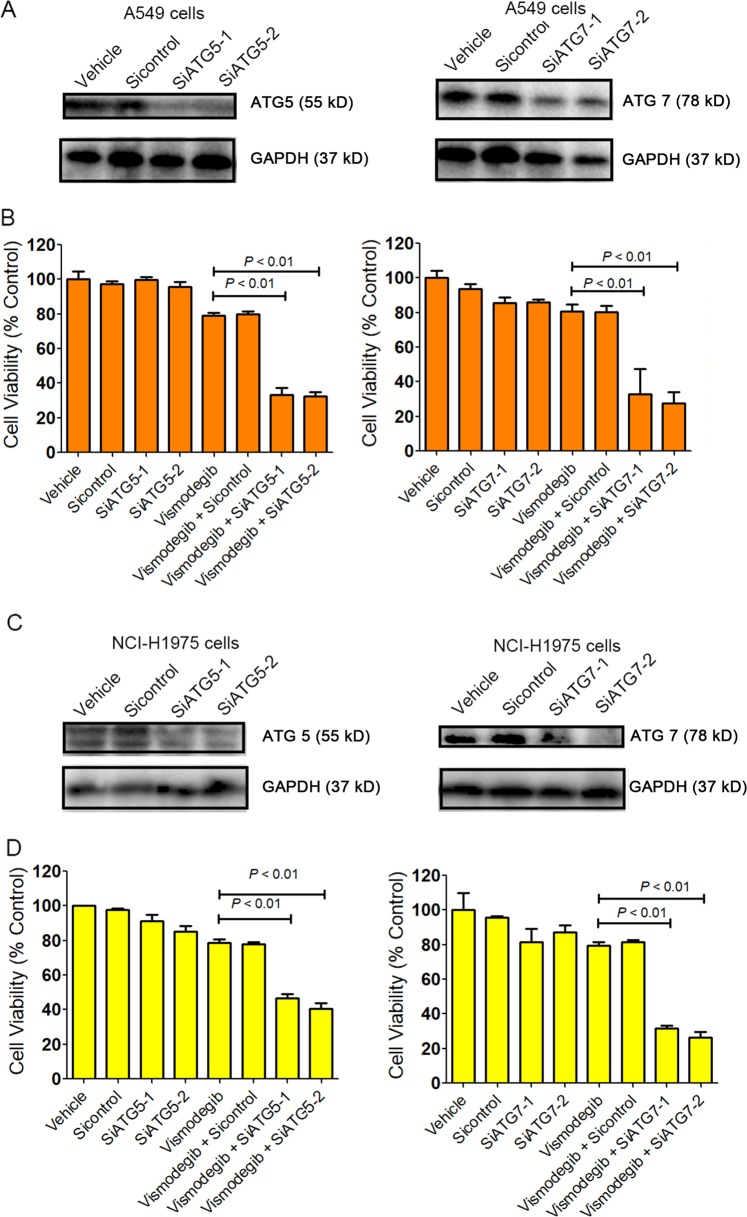
Enhanced cytotoxic effect of vismodegib in LUAD cells after autophagy blockage. **a**, **c** Autophagy blockage by siRNAs against ATG5 and ATG7. Cells were transfected with ATG5 and ATG7 siRNAs for 48 h and then lysed for western blot analysis. Sicontrol is a sequence that anti-luciferase and is used as a negative control. **b**, **d** Enhanced cytotoxic effect of vismodegib in LUAD cells after ATG5 and ATG7 knockdown. The MTT assay has been repeated for three times

As autophagic flux was triggered after A549 and NCI-H1975 cells were exposed to vismodegib, we postulated that autophagy might contribute to the resistance to vismodegib therapy in LUAD cells. siRNAs against ATG5 and ATG7 were employed to downregulate the activity of autophagy in A549 and NCI-H1975 cells. As illustrated in Fig. 4a, c, the expression of ATG5 and ATG7 was impaired after gene silencing by RNA interference, when compared with the vehicles. As ATG5 and ATG7 are both required for autophagosome-related double-membrane formation^26^, our results demonstrated that the activity of autophagy was suppressed after ATG5 and ATG7 knockdown. Then, the cell viabilities of A549 and NCI-H1975 cells were assessed after autophagy was blocked by the siRNAs. MTT assay showed that inhibition of autophagy by knocking down ATG5 and ATG7 significantly enhanced the cytotoxic effect of vismodegib on A549 and NCI-H1975 cells (Fig. 4b, d). Therefore, our data indicated that inhibition of autophagy could rescue the sensitiveness of A549 cells and NCI-H1975 cells to vismodegib therapy, indicating that inhibition of autophagy could facilitate cytotoxic effect of the Hedgehog pathway suppression in LUAD cells.

### Enhanced antitumor effect of Shh signaling blockage in A549 and NCI-H1975 xenograft models after autophagy inhibition

**Fig. 5:**
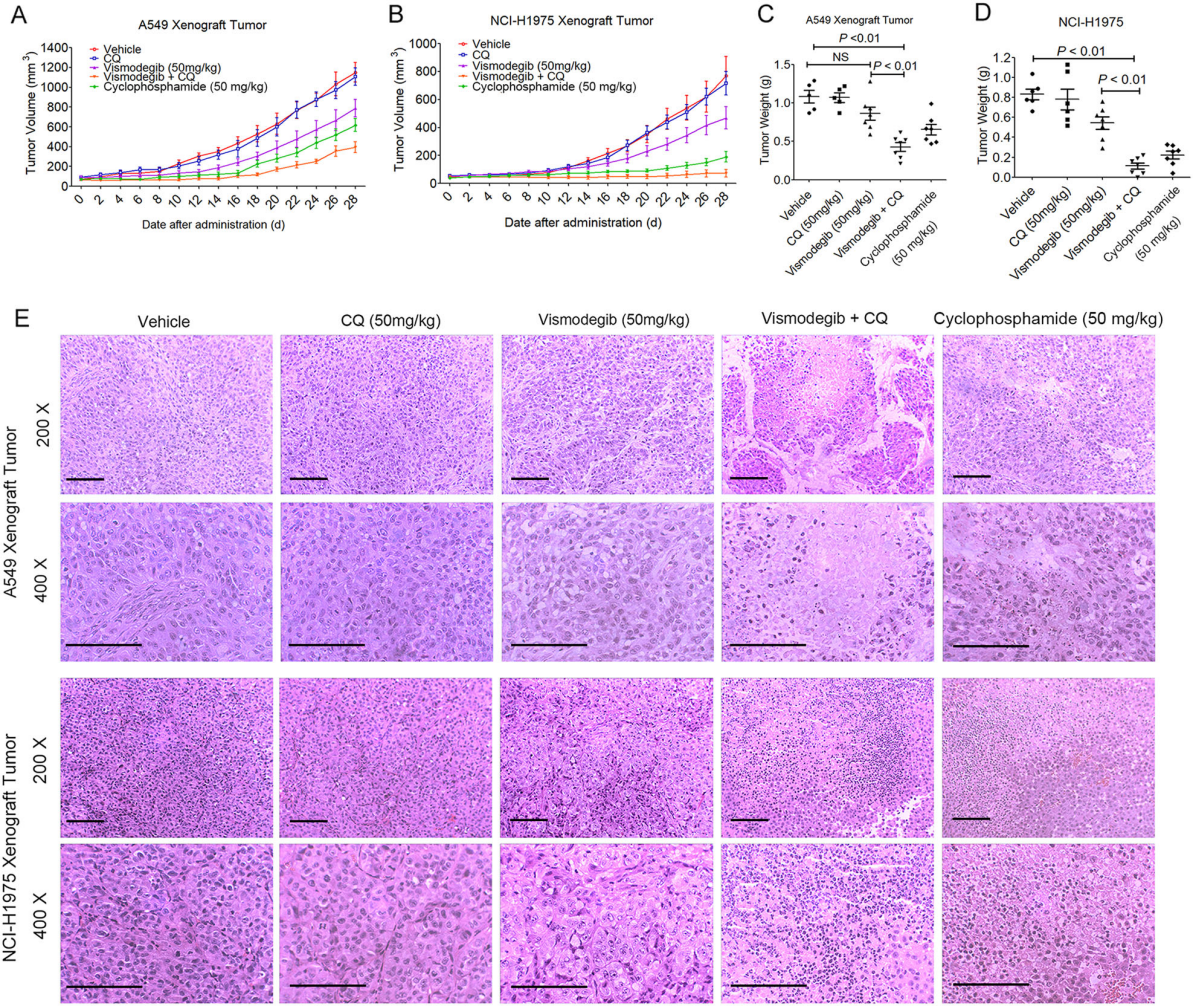
Rescued sensitiveness of LUAD xenograft tumors to vismodegib therapy in nude mice. **a**, **b** Tumor growth curve of A549 and NCI-H1975 xenograft tumor during 28 days post the treatment of vismodegib combined with/without autophagy inhibitor. **c**, **d** reduced tumor weight after mice treated with vismodegib and chloroquine. **e** H&E staining of the A549 and NCI-H1975 xenograft tumors from different group. Scale bar = 100 μm. The pharmacodynamic experiment to determine the anti-LUAD efficacy of vismodegib and chloroquine has been repeated once

The role of autophagy in the resistance against vismodegib therapy in LUAD cells were further evaluated in A549 and NCI-H1975 xenograft models. We challenged whether blocking autophagy could benefit the vismodegib-based therapy against A549 and NCI-H1975 transplanted tumors. Our results showed that treating mice with both Shh inhibitor vismodegib and autophagy suppressor chloroquine triggered a significant growth disadvantage of the xenograft tumors (Fig. 5a, b), indicating that autophagy inhibitor chloroquine could help vismodegib to restrict growth of LUAD in vivo. After 28-day observation, mice with xenografts were killed, and the tumors were then weighted (Figs. S6, S7). Mean tumor weight of the cohort co-treated with vismodegib and chloroquine were 482.61 ± 147.87 mg in A549 xenograft mice, and 112.91 ± 83.61 mg in NCI-H1975 xenograft mice. In contrast, mean tumor weight of the cohort of vehicle, chloroquine, vismodegib alone was 1082.75 ± 184.92 mg, 1072.57 ± 138.85 mg, and 864.32 ± 255.50 mg in A549 xenograft mice, and 833.33 ± 129.30 mg, 781.70 ± 254.70 mg, and 544.32 ± 164.47 mg in NCI-H1975 xenograft mice, respectively (Fig. 5c, d). Furthermore, H&E staining analysis showed the intensive necrotic LUADs tissues in mice treated with vismodegib plus chloroquine, while the vehicles and those treated with CQ or vismodegib alone exhibited no significant necrosis in LUADs tissues (Fig. 5e). These results suggested that A549 and NCI-H1975 xenograft tumors acquired resistance against vismodegib therapy in vivo, which could be attenuated by autophagy inhibition, indicating that blocking autophagy could facilitate therapeutic efficacy of the Hedgehog pathway inhibition on LUAD.

### ROS and caspase 3-dependent anti-LUAD effect of vismodegib after autophagy blockage

**Fig. 6:**
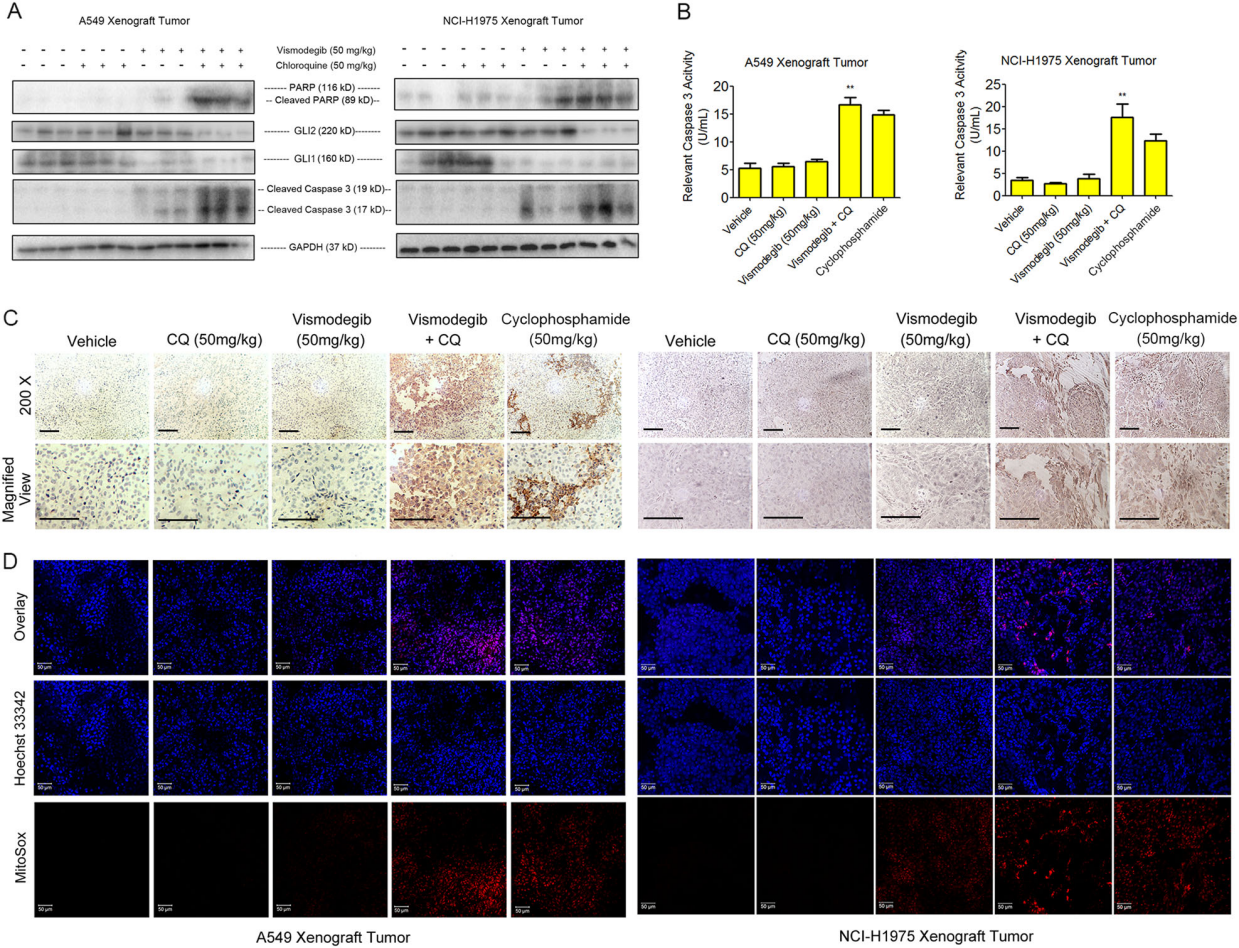
Rescued sensitivity of vismodegib in LUAD therapy by autophagy depletion through inducing apoptosis, ROS as well as inhibition of GLI2. **a** Reduced activity of GLI1 and GLI2 but activated caspase 3 and PARP in vismodegib-treated tumor-bearing mice after autophagy inhibition. **b** Enhanced activity of caspase 3 after autophagy inhibition. **c** Increased expression of cleaved caspase 3 in tumor necrotic area. Scale bar = 100 μm. **d** Production of ROS in LUAD tissue after treatment with vismodegib and chloroquine. Both western blots and ROS detection have been performed for three times

To further investigate the role of autophagy in vismodegib resistance in LUAD therapy, the apoptosis in LUAD tumor were assessed. Western blot analysis showed that apoptosis-related proteins caspase 3 and PARP were cleaved and activated in LUAD tissues from mice treated with both vismodegib and chloroquine (Figs. 6a; S8), while tumor from mice treated with saline, chloroquine, and vismodegib alone exhibited limited activated PARP or caspase 3. Furthermore, the histopathologic analysis showed that cleaved caspase 3 could be observed in cell nuclear in the necrotic LUAD tumor tissues, and activation of caspase 3 could be detected in the tumors from mice treated with vismodegib and chloroquine (Fig. 6b, c). Moreover, the role of caspase 3 in enhanced anticancer efficacy of simultaneous inhibition of autophagy and shh signaling was confirmed by application of Z-VAD-fmk, a caspase inhibitor. We found that suppression of caspase 3 cleavage by Z-VAD-fmk led to the rescued viabilities of A549 and NCI-H1975 cells that were exposed to vismodegib and chloroquine (Fig. S9). Therefore, our results indicated caspase 3-dependent apoptosis was involved in vismodegib-induced anti-LUAD effect after autophagy suppression.

Reactive oxygen species (ROS) is a critical mediator for cell death and apoptosis, which was related to the activation of caspase-dependent signaling. MitoSox dye was employed to assess the mitochondrial damage and mitochondrial-related ROS production. As shown in Fig. 6d, LUAD tissues from the cohort of vehicle, chloroquine, and vismodegib showed no significant ROS fluorescence under the confocal microscope, while co-treatment with vismodegib and chloroquine elicited a remarkable ROS production in LUAD tumors in vivo.

Therefore, our results indicated that ROS and caspase 3 were probably involved in the anti-LUAD effect elicited by vismodegib plus chloroquine.

### Downregulated expression of GLI2 in A549 and NCI-H1975 xenograft tumors after vismodegib and autophagy inhibitor exposure

It was reported that the Shh signaling pathway was related to tumor growth and survival^27^, but its correlation with autophagy was still unclear. Indeed, the Shh inhibitor vismodegib could efficiently inhibit the expression of GLI1, but not GLI2. Importantly, knockdown of GLI2 level resulted in enhanced anti-LUAD effect of vismodegib in A549 and NCI-H1975 cells (Fig. S10), indicating the critical role of GLI2 in the therapy resistance against vismodegib in LUAD cells. Thus, we challenged if inhibition of autophagy could facilitate the inhibition of GLI2 activity. Western blot results revealed that either vismodegib alone or combined use of vismodegib and chloroquine could effectively inhibit the expression of GLI1. However, only co-treatment with vismodegib and chloroquine promoted the inhibitory effect on GLI2. In comparison, treatment with saline, chloroquine, and vismodegib did not have notable effect on the GLI2 (Fig. 6a). Therefore, our results showed that inhibition of autophagy contributed to the GLI2 inhibition induced by vismodegib in vivo, which indicated that inhibition of autophagy could sensitize the LUAD xenograft tumors to the Shh inhibitors therapy through suppression of GLI2.

## Discussion

As one of the most common tumors in respiratory system, LUAD comprises over 50% of all cases of NSCLC^28^. Although tyrosine kinase inhibitors have saved a part of patients with EGFR mutant LUADs, many people are still suffered from the disease because of the primary and acquired therapy resistance. Previous researches and our preliminary results have demonstrated that the Shh signaling pathway is upregulated in LUAD. Since the Shh signaling pathway played a key role in tumor growth and tumor progression^29,30^, inhibitors of the Shh pathway theoretically had the antitumor effects on LUADs. However, our results showed that the inhibitor of the Shh pathway, vismodegib, exhibited only minor impact on the LUAD cell growth. In this study, we evaluated the potential of vismodegib in LUAD therapy and discussed the underlying mechanism of resistance of LUAD to vismodegib treatment.

The activity of the Shh signaling pathway was assessed in LUAD cells after vismodegib treatment. Our data revealed that vismodegib only reduced GLI1 and SMO transcriptionally, but had no significant effect on GLI2. However, GANT61, a direct inhibitor of GLI2, was demonstrated to have appropriate anti-NSCLC efficacy in vitro and in vivo^31^, indicating that the GLI2 inhibition seemed to be related to the anti-LUAD effect of Shh inhibitors. Since it was well documented that GLI2 played a much more important role than GLI1 and SMO did in the therapy, prognosis, and metastasis of LUAD^32,33^, we supposed that the failed modulation of GLI2 is one of the probable reasons that resulted in therapy resistance to vismodegib in LUAD.

Actually, the expression of GLIs could be regulated through a SMO-independent pathway. A series of factors such as oncogenic receptor tyrosine kinases, RAS/MAP kinase, PI3K/AKT/S6K, DYRK1A, PKC, TGF-β, and histone deacetylases could enhance the transcriptional activity of GLIs in human cancer cells^34,35^. It was well documented that TGF-β always acted as a key player in epithelial–mesenchymal transition (EMT)-mediated tumor metastasis and therapy resistance in LUADs, which might be the likely mediator to modulate the transcription of GLIs^36^. However, our data showed that the expression level of TGF-β exhibited no significant change in A549 and NCI-H1975 xenograft tumors after vismodegib treatment. Therefore, our results indicated that GLI2 was regulated in LUADs through TGF-β-independent way.

Autophagy is a main factor that caused primary or acquired resistance in cancer therapy. It was usually regarded as a cyto-protective mechanism when cancer cells were facing drug treatment or growth disadvantage^13^. Actually, regulation of the Shh pathway was associated with autophagy initiation. It was reported that inhibition of GLI1/2/3-related signaling transduction will lead to the initiation of autophagy in the liver, pancreatic, B-NHL, and CML cells^9–11,21^. Here, we reported that after treatment with vismodegib, the mRNA level of ATG5 and ATG7 reached a rather high level in LUAD cells. Furthermore, the upregulated autophagy was confirmed by enhancement of punctuate fluorescence, appearance of membrane-associated protein LC3-II, formation of characteristic autophagosomes, and autophagosomes fusion into lysosomes in LUAD cells after blockage of the Shh pathway. Therefore, our results suggested that suppression of the Shh pathway triggered abnormal autophagic initiation and autophagic flux in LUAD cells.

Previous researches suggested that autophagy is important for cancer therapy, and modulation of autophagy could be a therapeutic approach to benefit particular drug treatment, or to combat the therapy resistance. In the past few years, the role of autophagy in vismodegib-based therapy proved to be controversial. It was reported that vismodegib-induced autophagy tended to play a cyto-protective role and probably triggered the therapy tolerance in B-cell lymphoma cells and chronic myeloid leukemia cells^10,11^, while inhibition of autophagy in HepG2 and Hep3B cells impaired the cytotoxicity induced by vismodegib^21^. Thus, autophagy was likely to act as a double-edged sword in Shh inhibitor-based cancer therapy. In this study, we used both the pharmaceutical inhibitor chloroquine and siRNAs for autophagy blockage after vismodegib treatment. We found that knockdown of ATG5 and ATG7 significantly sensitized the A549 and NCI-H1975 LUAD cells to the vismodegib treatment while inhibition of autophagy by chloroquine helped to overcome the therapy resistance of xenograft tumors to vismodegib via eliciting cytotoxic ROS generation and caspase 3-dependent apoptosis in vivo. Therefore, our data indicated that autophagy might be a probable mechanism responsible for resistance of LUAD to vismodegib Hedgehog inhibitor-based therapy.

It was documented that inhibition of the Shh pathway led to the increased activity of autophagy. However, whether modulation of autophagy could regulate the activity of the Shh signaling pathway still remained unclear. Here, we reported for the first time that inhibition of autophagy in vivo could contribute to the suppression of GLI2, and thereby facilitated the sensitiveness of LUAD to the vismodegib therapy. Therefore, our results suggested that autophagy might interplay with the GLI2, which acted as a key player in therapy resistance against the Shh pathway suppression in LUAD treatment.

**Fig. 7:**
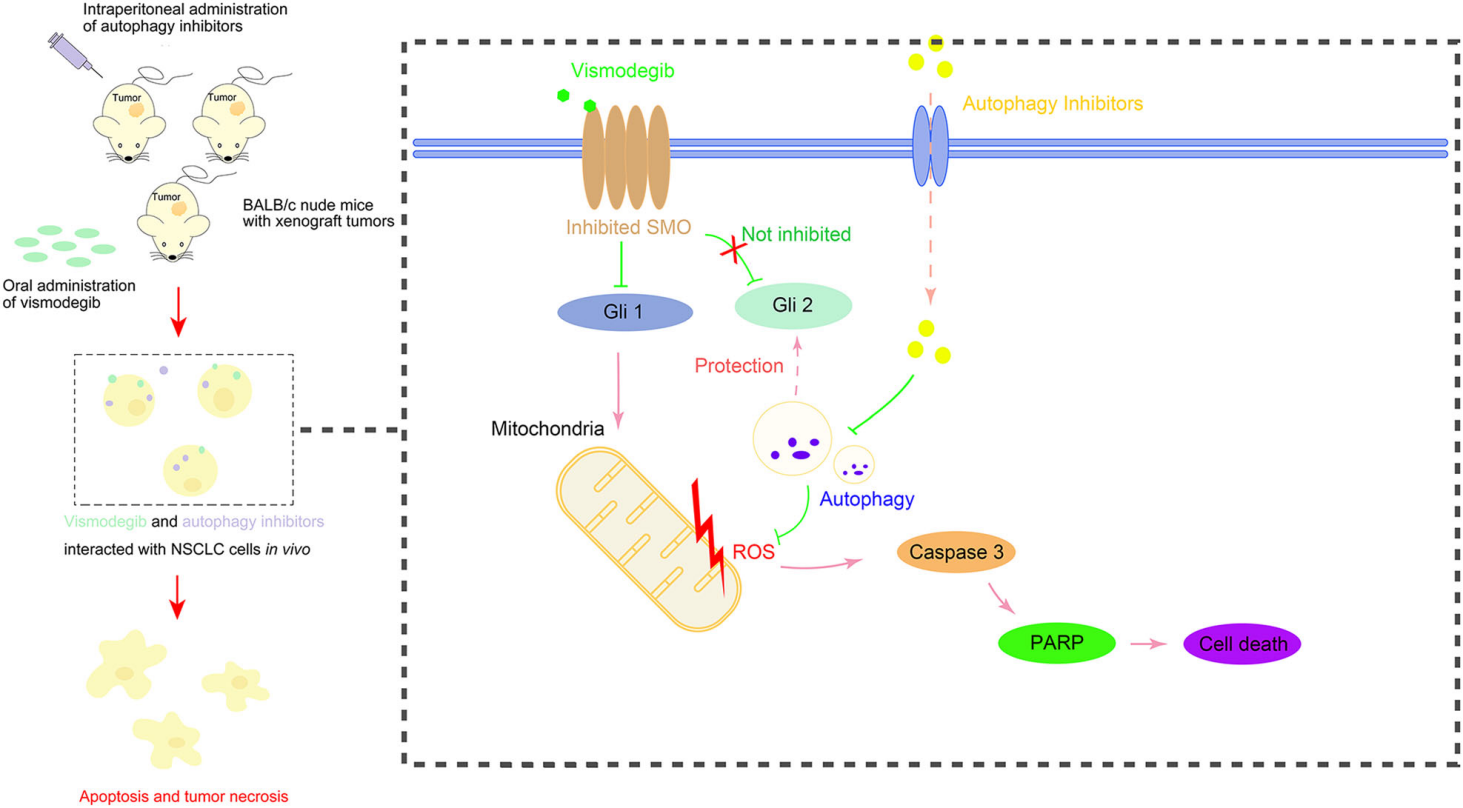
Autophagy triggers the therapy resistance against to Shh pathway inhibition in LUAD treatment

In conclusion, this research evaluated the potential of inhibiting the Shh signaling pathway in LUAD treatment and evaluated the mechanism why the LUAD cells were resistant to the Shh inhibitor-based therapy (Fig. 7). Our data exhibited that the Shh signaling pathway was activated in A549 and NCI-H1975 LUAD cells, while vismodegib, a Shh inhibitor, showed minor anti-LUAD efficacy in vitro and in vivo. During this process, autophagy was triggered as a response to the Shh pathway inhibition, which could be characterized by three main stages of autophagic flux, including autophagosomes formation, fusion of autophagosomes into lysosomes, and degradation of lysosomes in autophagosomes. Importantly, downregulating autophagy by siRNA against ATG5 and ATG7 rescued the sensitivity of A549 and NCI-H1975 LUAD cells to vismodegib in vitro, while the pharmaceutical inhibitor of autophagy, chloroquine, sensitized LUAD to the vismodegib therapy in vivo via facilitating the overproduction of ROS, acceleration of apoptosis and suppression of GLI2. Our data revealed that inhibition of autophagy promoted the sensitiveness of LUAD to the Shh inhibitor and highlighted a potential approach for LUAD therapy via simultaneous targeting the Shh signaling and autophagy pathway.

## Materials and methods

### Cell lines and culture

Human LUAD A549 and NCI-H1975 cell lines were obtained from Cell Bank of Chinese Academy of Sciences (Shanghai, China) and maintained in the RPMI-1640 medium containing 10% of fetal bovine serum (FBS) (Invitrogen, San Diego, CA, USA), 2 mM L-glutamine and 1% penicillin–streptomycin at 37 °C in a humidified incubator with 5% CO_2_. Cells were authenticated by the Cell Bank of Chinese Academy of Sciences, and were passaged for fewer than 6 months after receipt form cell bank or resuscitation.

### Reagents

Vismodegib was purchased from Shanghai BiochemPartner Co., Ltd (Shanghai, China) and dissolved in dimethyl sulfoxide at the concentration of 30 mM. Cyto-ID^®^ Autophagic Detective Kit was obtained from Enzo Biochem, Inc. (Farmingdale, NY, USA). Chloroquine (CQ) was purchased from Sigma (St Louis, MO, USA). The primary antibodies against LC3B, GLI1/GLI2/SMO, GAPDH and TGF-β were obtained from Cell Signaling Technology (Danvers, USA), and the secondary antibody peroxidase-conjugated affiniPure goat anti-rabbit IgG were from Abcam (San Diego, USA). The detection kit for caspase 3 activity was purchased from Beyotime (Jiangsu, China). MitoSOX was obtained from Invitrogen™ (Grand Island, NY).

### Real-time PCR

The extraction of mRNA, reverse transcription, and quantitative real-time PCR were performed as previously indicated^37^. RNA was extracted using TRIZOL (Invitrogen, USA) and quantified with NanoDrop Spectrophotometer (Thermo Scientific, Waltham). RT‐qPCRs experiments were performed in a CFX384 thermocycler (Bio‐Rad, Milan, Italy) using 10 ng/reaction of cDNA as a template and SYBR Fast Universal Ready Mix Kit (TOYOBO, Japan). The data were analyzed using Bio‐Rad CFX manager software (Bio‐Rad, Milan, Italy). Primers were synthesized by Sangon Biotech (Shanghai, China). The related mRNA level was normalized by the level of GAPDH. The primers used in this study were as follows:

**Table:** 

Gene	Forward primer	Reverse primer
GLI1	AGCGTGAGCCTGAATCTGTG	CAGCATGTACTGGGCTTTGAA
SMO	TGCCACCAGAAGAACAAGC	GGAGATCTCTGCCTCAACCA
GLI2	TGGACGTGTCCCGTTTCTCC	CCACTAGCGAGTTGGGTGAG
ATG5	GCTTCGAGATGTGTGGTTTG	CAGTGGTGTGCCTTCATATT
ATG7	ACCCAGAAGAAGCTGAACGA	CTCATTTGCTGCTTGTTCCA

### siRNA transfection

siRNA against ATG5, ATG7, GLI2, and a negative control were purchased from Guangzhou Ribo Bio Co., Ltd (Guangzhou, China). According to the instructions provided by manufacturer, A549 and NCI-H1975 LUAD cells (2 × 10^5^ cells/mL) were transfected the siRNA oligonucleotides by using Lipofectamine 2000 Transfection Reagent (Invitrogen™, San Diego, USA), followed by incubation for 48 h for further treatments or western blot.

### Thiazolyl blue tetrazolium bromide (MTT) assay

The viability of LUAD cells was determined by the MTT-based assay. Approximately 1 × 10^4^ cells were seeded in each well, and then treated with/without vismodegib or/and autophagy inhibitors. Then, MTT solution was added into each well and interacted with LUAD cells for 1 h at 37 °C, followed by the measurement of optical density (O.D.) at 570 -nm absorbance wavelength.

### Western blot analysis

Cells were washed with cold phosphate buffer saline (PBS) and then lysed in radio immunoprecipitation assay buffer (RIPA) for 30 min on ice. The lysates were discarded after a 12000×*g* centrifugation at 4 °C for 3 min. Protein content was measured by Bicinchoninic Acid-Based Protein Quantification Kit (Biotech Well, Shanghai, China), and 50 μg of each protein sample was re-suspended in 4 × loading buffer, maintained at boiling water for 10 min, resolved by electrophoresis on 12% SDS-based polyacrylamide gel electrophoresis (SDS-PAGE), and transferred to polyvinylidene fluoride (PVDF) membranes. The reacted membranes were washed in tris-buffered saline solution (TBS) for three times, followed by its immediately blocked in tris-buffered saline solution with 0.1% Tween-20 (TBST) containing 3% bovine serum albumin for 1 h. Then the PVDF membranes were incubated overnight with antibodies (1:1000 of dilution) in TBST buffer at 4 °C overnight and washed in TBST for three times and hybridized with horseradish peroxidase-conjugated anti-rabbit antibody for 1 h. Afterwards, the immunoreactive bands were detected by chemiluminiscence reagent (Pierce Biotechnology, Inc, USA). Blots were re-probed with antibodies for GAPDH and used as internal control for protein loading and transfer. Densitometric values of protein bands were quantified by the IQuantTL software (GE Healthcare, England, UK).

### Animals

Animal care were carried out according to the National Institutes of Health Guide for the Care and Use of Laboratory Animals after the experiments were approved by Animal Ethical Committee, School of Pharmacy, Fudan University.

The experiments were performed on adult male BALB/c nude mice with its weight at 20.0 ± 2 g. Animals were maintained in a specific pathogen-free room at temperature of 22 °C ± 0.5 °C on a 12 h light–dark cycle (8:00 a.m.–8:00 p.m.), with free access to food and water.

### Xenograft tumor model assay

To establish the subcutaneous LUAD xenograft model, BALB/c nude mice were subcutaneously injected with 5 × 10^6^ of A549 or NCI-H1975 cells suspended in the RPMI-1640 medium containing 50% Matrigel^®^ Matrix (Coining, China). After random assignment, tumor-bearing mice were treated with vismodegib (50 mg/kg, every other day, oral administration), saline (100 μL, both oral administration and intraperitoneal injection), chloroquine (50 mg/kg, once every day, intraperitoneal injection), both vismodegib and chloroquine or cyclophosphamide (50 mg/kg, oral administration, every other day). After corresponding treatment for 28 days, the mice were killed for tumor collection. During the period, the tumor volumes were measured and calculated.

### Confocal fluorescence

LUAD cells were co-incubated with 30 μM of vismodegib for indicated time, and the tissue samples were prepared immediately in PBS buffer after the mice were killed. Cells and tissues were disposed with the Cyto-ID^®^ Autophagic Detective Kit, MitoSox Dye, Lysotracker DND99 Dye, as described^38,39^.

### Transmission electron microscopy

LUAD cells were treated with 30 μM of vismodegib for 36 h, while tissues were prepared in 4% of paraformaldehyde aqueous solution after the nude mice were killed. Then, cells or tissues were collected and prepared as mentioned^40^. Samples were analyzed by a JEM 1400 plus transmission electron microscope (JEOL, Japan, Inc.).

### Statistics analysis

Statistics analysis was performed with SPSS 17.0. The results were expressed as means ± SEM. Comparisons were performed using Student’s *t* test (two tailed), and *P*-value < 0.05 was regarded as statistically significant.

## Supplementary information

Supplemental Figures
