# Supplementary material for: Regulating autophagy facilitated therapeutic efficacy of the sonic Hedgehog pathway inhibition on lung adenocarcinoma through GLI2 suppression and ROS production
Source: Cell Death Dis. 2019 Aug 19;10(9):626. doi: 10.1038/s41419-019-1840-6 (PMC6700102; doi:10.1038/s41419-019-1840-6)

Cell Death & Disease

Supporting Information

Regulating autophagy facilitated therapeutic efficacy of sonic hedgehog pathway inhibition on lung adenocarcinoma through GLI 2 suppression and ROS production

Correspondence:

Xilin Sun:

TOF-PET/CT/MR Center, The Fourth Hospital of Harbin Medical University, 766 Xiangan N street, Harbin, Heilongjiang 150028, China. sunxl@ems.hrbmu.edu.cn

Baozhong Shen:

Molecular Imaging Research Center, Harbin Medical University, Harbin, TOF-PET/CT/MR center, The Fourth Hospital of Harbin Medical University, Harbin, Heilongjiang, China. shenbz@ems.hrbmu.edu.cn

Dianwen Ju:

Minhang Hospital, Fudan University, 170 Xinsong Road, Shanghai 201199, China. Department of Microbiological and Biochemical Pharmacy & The Key Laboratory of Smart Drug Delivery, Ministry of Education, School of Pharmacy, Fudan University, Shanghai, 201203, China

Figure S1 Upregulated GLI1 expression in LUAD cells after SMO activation. Cells were treated with/without SAG for 24 h, followed by extraction of the mRNA. The GLI1 mRNA level was normalized by the level of GAPDH * $P < 0.05$ v.s. Vehicle; ** $P < 0.01$ v.s. Vehicle. The experiments were repeated for 3 times.

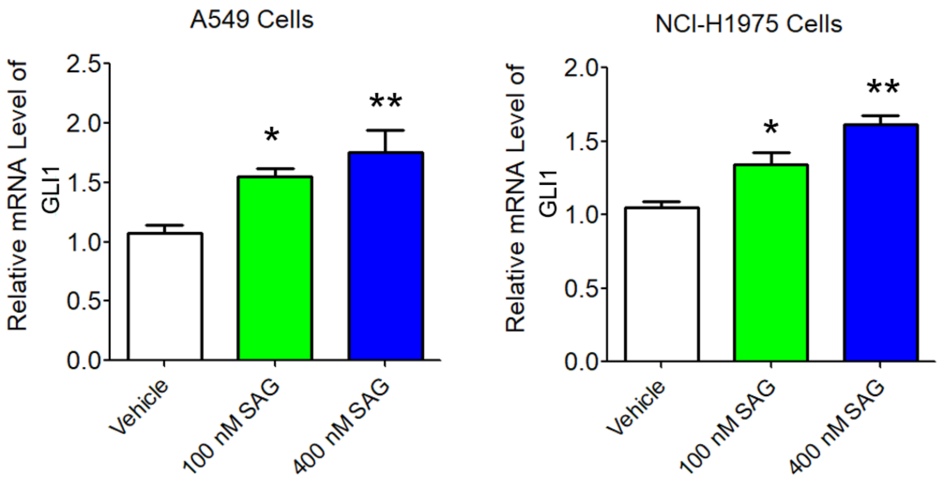

Figure S2 Statistical analysis of related protein level in Figure 2D (n = 6)

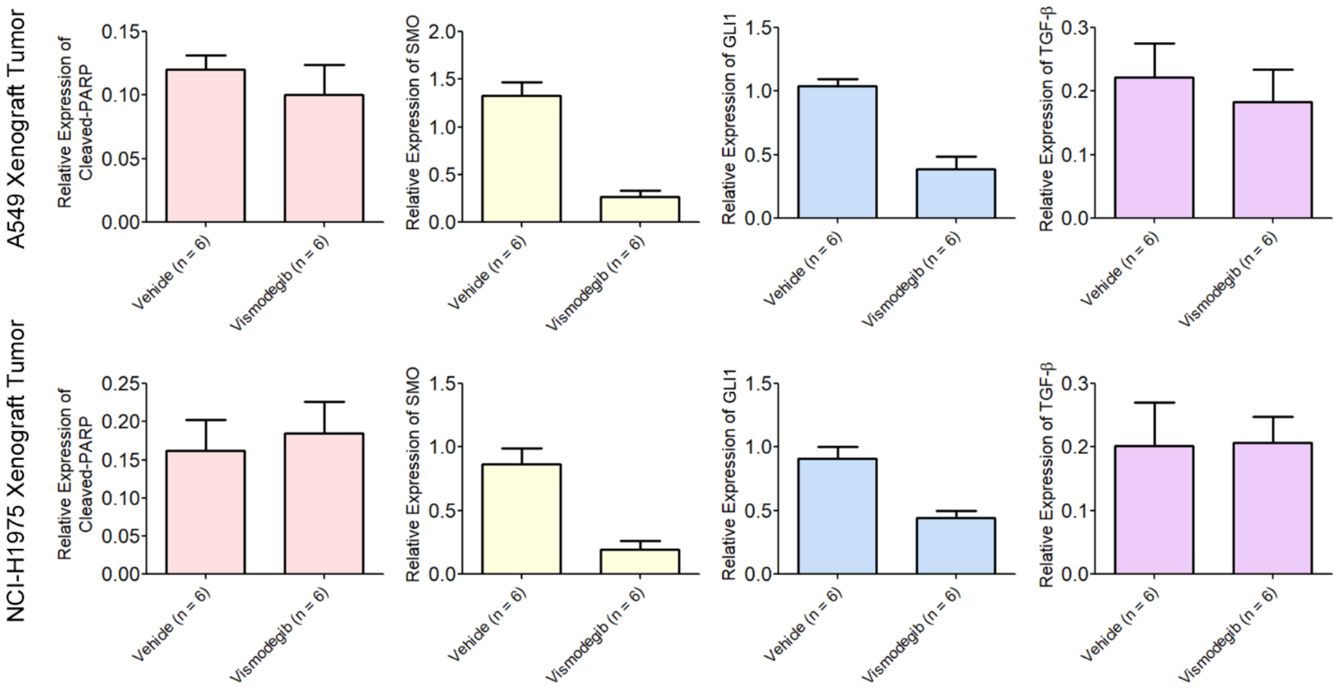

Figure S3 Enhanced transcription of ATG5 and ATG7 in A549 and NCI-H1975 cells after vismodegib treatment. A549 Cells (A) and NCI-H1975 cells (B) were lysed by Trisol and prepared for Realtime-PCR detection after vismodegib exposure (n = 3). The experiments were performed for 3 times.

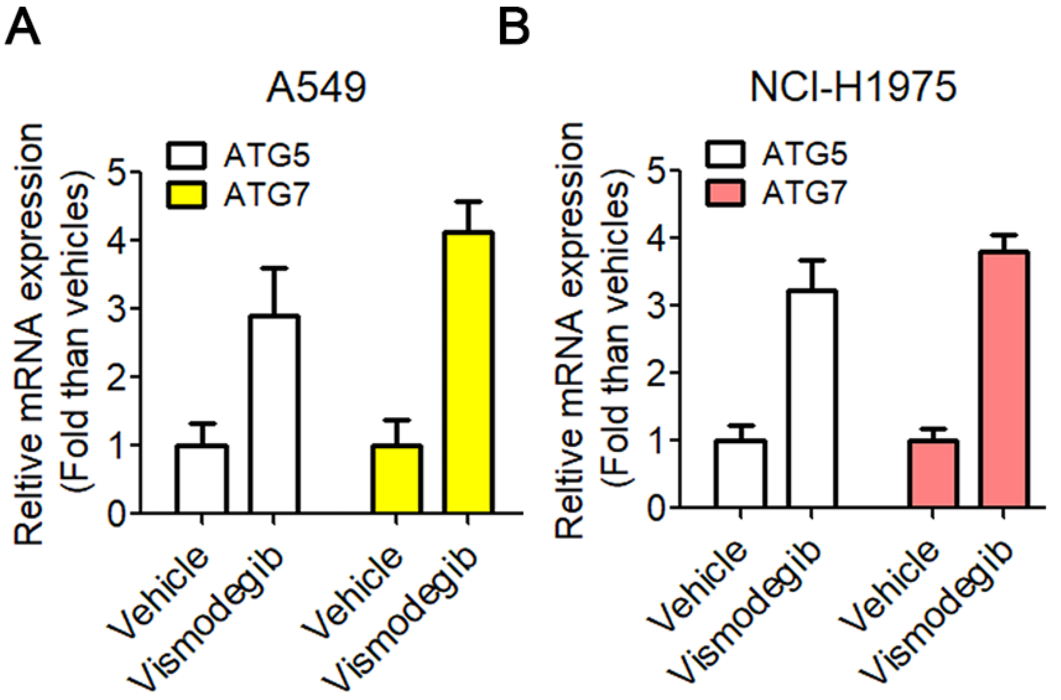

Figure S4 Autophagosomes formation *in vitro* and *in vivo* after vismodegib therapy

A, B: Suppression of GLI 1 expression and increased LC3-II level after cells exposure to vismodegib. C: Increased LC3-II expression in A549 and NCI-H1975 transplanted tumors. D: Vismodegib induced autophagic vesicles formation in A549 and NCI-H1975 cells. Cells were treated with 30 μM of vismodegib for 12h and 24 h. Then, cyto-ID green dye was applied to detect the autophagosomes formation in A549 and NCI-H1975 cells.

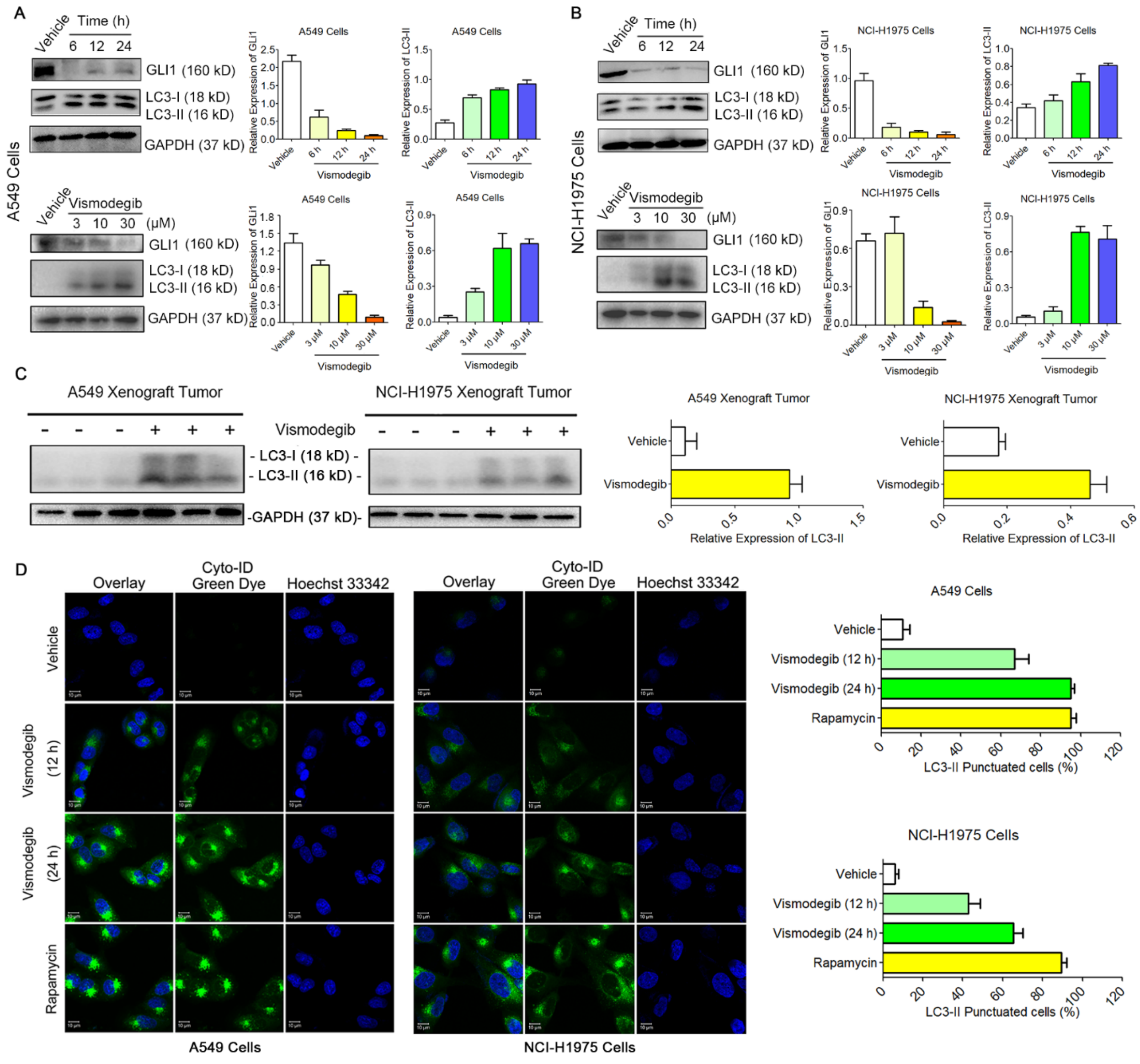

Fig. S5 Vismodegib induce neither growth inhibition (A, B) nor upregulated level of LC3-II (C) in HEK293 cells.

HEK293 cells were treated with different concentrations of vismodegib for 12-48 h. MTT assays were applied to detect the cytotoxic effect of vismodegib on HEK293 cells, and western blots were employed to monitor the change of LC3-II after vismodegib exposure. Both western blots and MTT assays have been repeated for 3 times

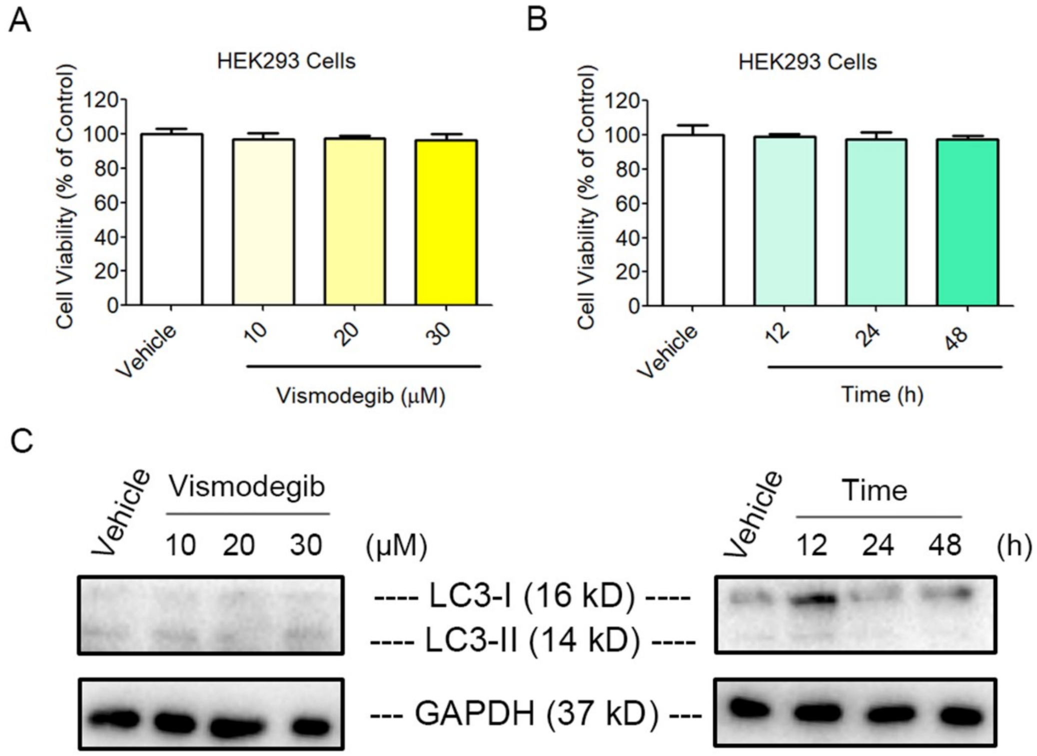

Figure S6 A549 xenograft tumors after mice were sacrificed after 28-day treatment.

A549 xenograft tumors were collected immediately when mice were sacrificed. (Cyclophosphamide: CTX)

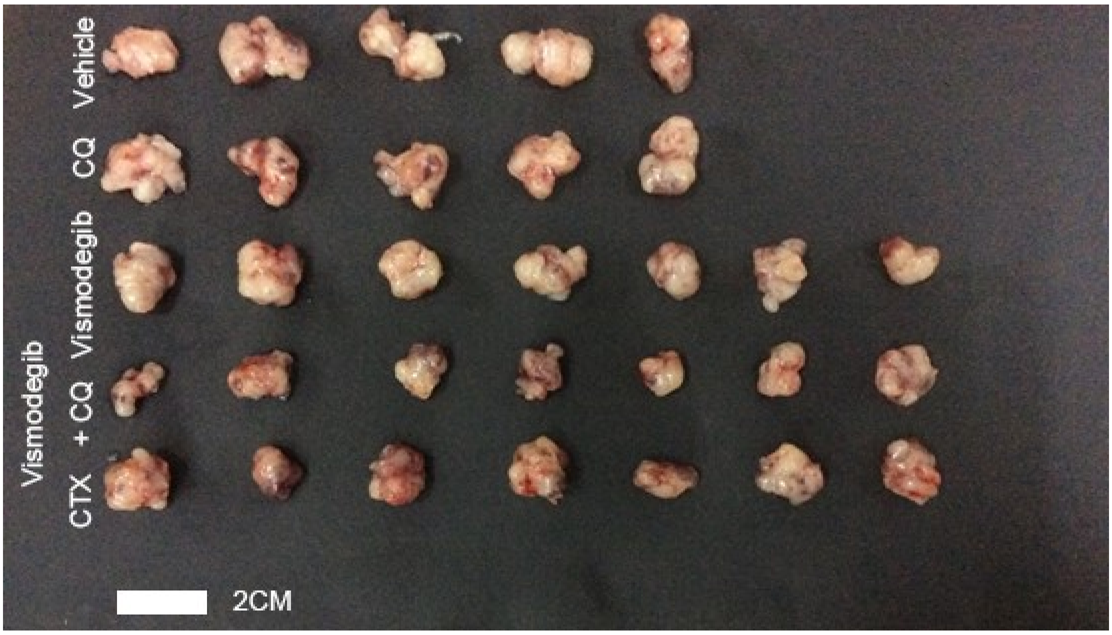

Figure S7 NCI-H1975 xenograft tumors after mice were sacrificed after 28-day treatment. NCI-H1975 xenograft tumors were collected immediately when mice were sacrificed. (Cyclophosphamide: CTX)

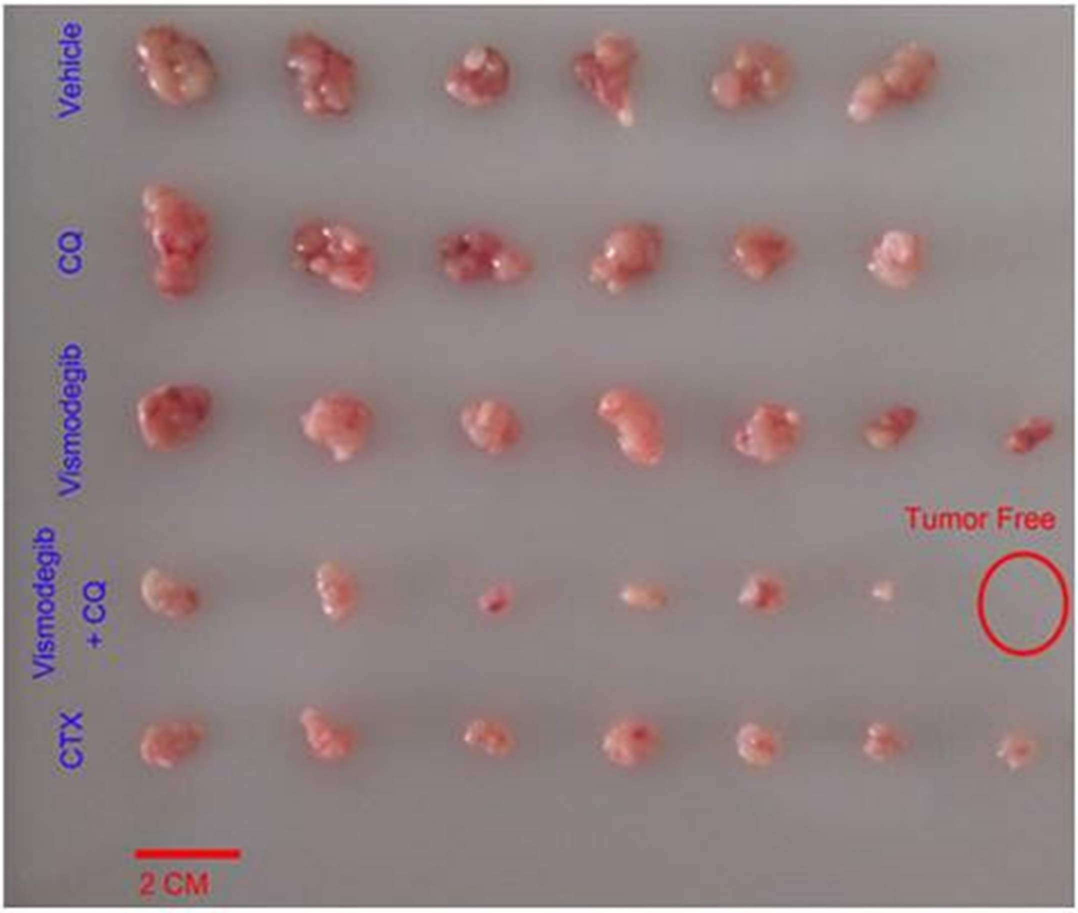

Figure S8 Statistical analysis of related protein level in Figure 6A (n = 5)

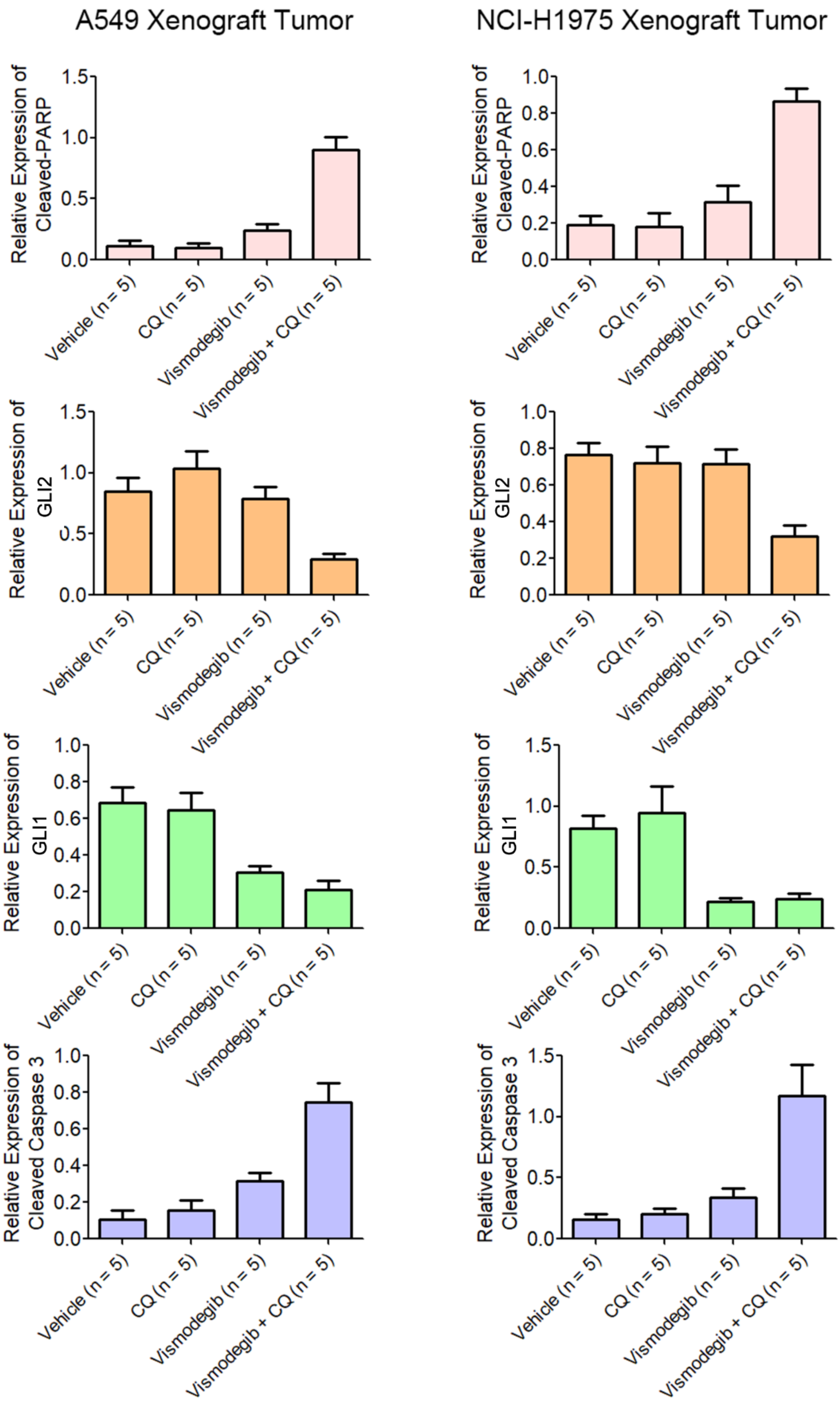

Figure S9. Caspase 3-dependent cytotoxic effect of vismodegib after autophagy blockage in A549 and NCI-H1975 cells.

Cells were treated with vismodegib, Z-VAD-fmk or chloroquine (CQ) for 72 h. The activation of Caspase 3 and the cell viability was measured by western blot and MTT assay, respectively. Both the western blot and the MTT assay have been repeated for 3 times.

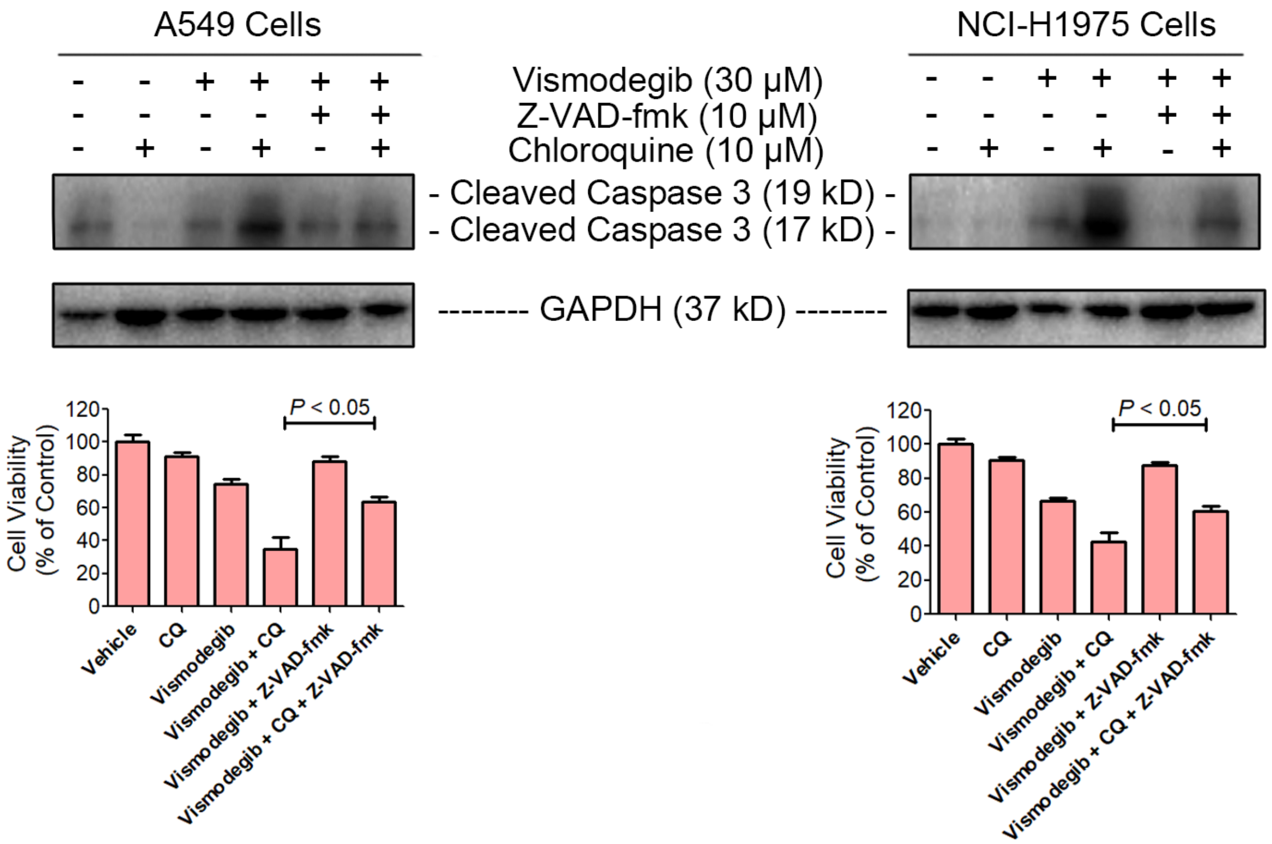

Figure S10 Knockdown of GLI2 promoted the anti-LUAD effect of vismodegib in A549 and NCI-H1975 cells

Cells were treated with anti-GLI2 siRNA for 48 h to silence the expression of GLI2, and then co-cultured with vismodegib for 72 h. The experiments were performed for 3 times.

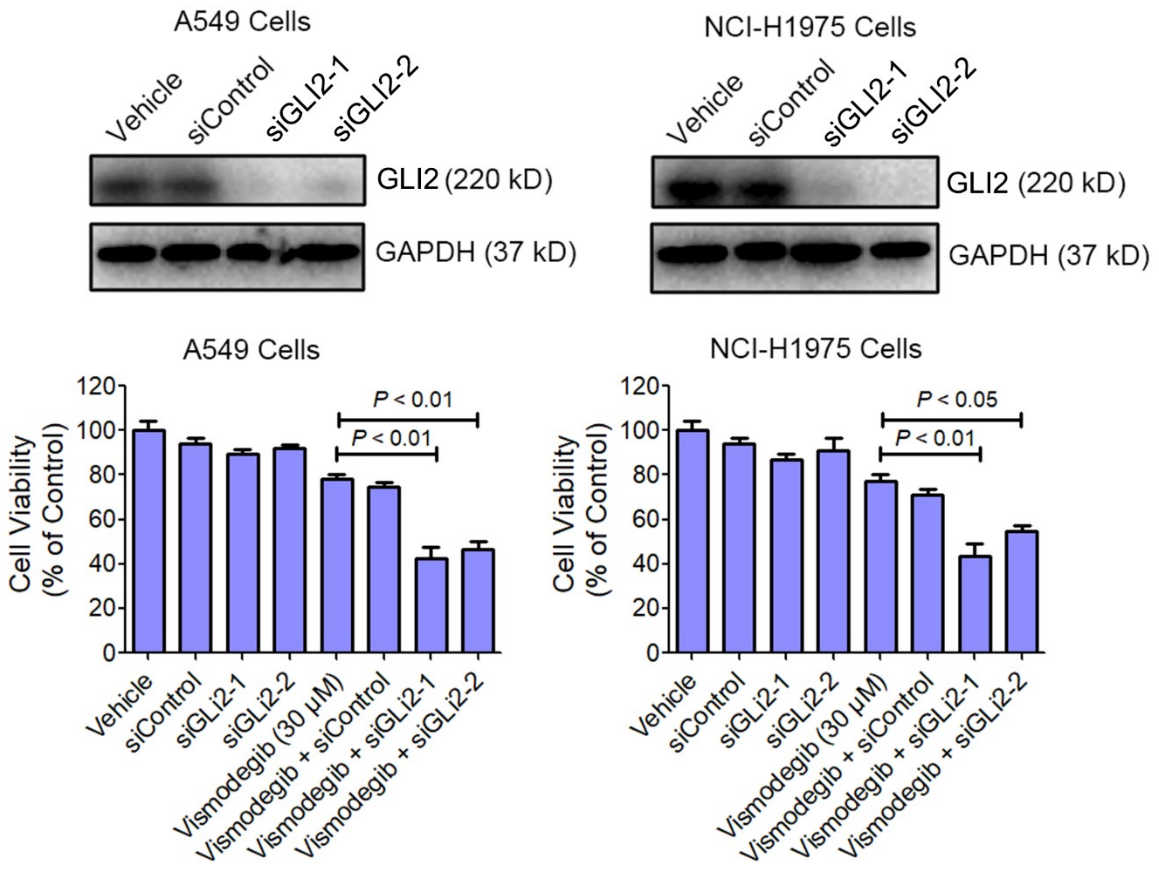

Figure S11. Repeated results of Fig. 1A, B (A), Fig. 2D (B), and Fig. 6A (C)

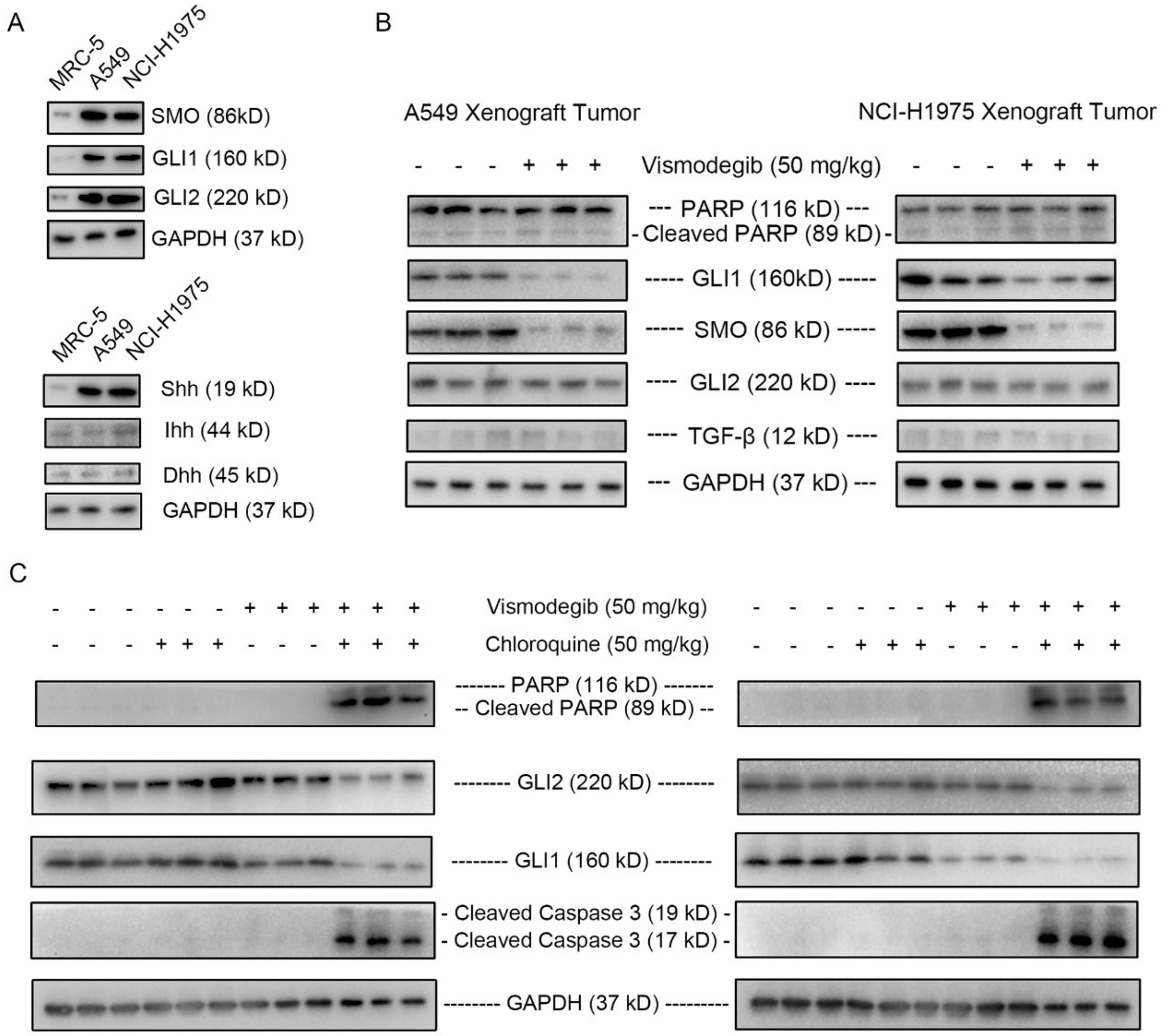

Supplement: Supplementary file 1 — Supplemental Figures [file 41419_2019_1840_MOESM1_ESM.pdf]
